# Comment on Kronenfeld et al. Clinical Outcomes for Primary and Radiation-Associated Angiosarcoma of the Breast with Multimodal Treatment: Long-Term Survival Is Achievable. *Cancers* 2021, *13*, 3814

**DOI:** 10.3390/cancers13225707

**Published:** 2021-11-15

**Authors:** Markus Notter, Emanuel Stutz, Andreas R. Thomsen, Attila Kollár, Peter Vaupel

**Affiliations:** 1 Radiation Oncology, Lindenhofspital Bern, 3012 Bern, Switzerland; 2 Member of the Swiss Hyperthermia Network, 5000 Aarau, Switzerland; Emanuel.stutz@insel.ch; 3 Department of Radiation Oncology, Inselspital, Bern University Hospital, University of Bern, 3010 Bern, Switzerland; 4 Department of Radiation Oncology, University Medical Center, University of Freiburg, 79106 Freiburg, Germany; andreas.thomsen@uniklinik-freiburg.de (A.R.T.); vaupel@uni-mainz.de (P.V.); 5 German Cancer Consortium (DKTK), Partner Site Freiburg and German Cancer Research Center (DKFZ), 69120 Heidelberg, Germany; 6 Department of Medical Oncology, Inselspital Bern University Hospital, University of Bern, 3010 Bern, Switzerland; attila.kollar@insel.ch; 7 Sarcoma Center, Inselspital Bern University Hospital, University of Bern, 3010 Bern, Switzerland

On 2 July 2021, Kronenfeld et al. [1] published an excellent analysis of the clinical outcomes of primary angiosarcomas of the breast (PAS) and radiation-associated angiosarcomas of the breast (RAASB) treated with multimodality therapy in the last decade (2010–2020). They concluded, that a multimodal treatment approach that included neoadjuvant chemotherapy (NAC), radiation therapy (RT) in selected patients, surgery (S), and adjuvant chemotherapy (AC) may result in improved outcomes, including prolonged survival. Despite the small sample size, they demonstrated that NAC, especially when associated with a pathologic complete response, may contribute to these substantial results. We thoroughly support their conclusion that in addition to dedicated surgical procedures as the primary means of treatment, further pre- or postoperative treatment is urgently needed. However, the recommendation for focusing predominantly on systemic treatments is too limited.

PAS and RAASB should be considered as different tumor entities with regard to treatment strategies largely due to the restricted treatment options for RAASB. PASs occur mostly in younger women and have a higher potential of distant metastasis. As RAASB patients have received prior breast cancer treatment with adjuvant radiation. With respect to PAS, aggressive NAC, postoperative AC, or full dosed postoperative RT could be considered, especially in cases with insufficient margins. This is less the case with radiation-associated angiosarcomas (RAAS). Previously irradiated sites do not always allow for an adequate re-irradiation (re-RT) dose. Surgical procedures should solve reconstructive challenges and may be hampered by prolonged wound healing among other complications. The effectiveness of systemic treatments can also be affected as a result of reduced perfusion in heavily pre-irradiated tissue. In addition, the incidence of local recurrences in both tumor types is rather high as reported by several authors [2]. Therefore, additional adjuvant local treatment as a therapeutic option must be evaluated as well.

In a similar timeframe as the aforementioned analysis (2011–2021), we have focused on new treatment strategies for RAASB specifically for recurrent tumors, which have the worst prognosis. Based on findings of earlier studies using hyperthermia and re-irradiation in locally recurrent breast cancer, we found that the combination of both modalities allowed for a reduction of the total re-irradiation dose to just 20 Gy using a hypofractionated schedule of 5 × 4 Gy once per week [3]. To our knowledge, this is the lowest total re-irradiation dose applied so far in a protocol that aims for effective tumor control. Contact-free water filtered infrared-A superficial hyperthermia allows for greater coverage of large-sized lesions, and can reduce the risk of thermal skin damage to a minimum [4]. In contrast to most reported protocols, our protocol involves the utilization of hyperthermia prior to re-RT. The low toxicity of this protocol even allows for repeat re-RT using the same dosage and schedule. This is crucial in the management of RAASB, which is often locally recurring despite the usage of large treatment fields and sufficient margins.

Coincidentally, our results [5] were published 4 days later after the study carried outby Kronenfeld et al. [1]. Both analyses obviously represented different RAASB situations in completely different tumor stages. The Kronenfeld cohort contained patients treated at first diagnosis in a localized stage as the clinical tumor size varied from 2 to 7 cm (median 2.8 cm). Therefore, all patients were resectable and in 100% a R0 resection was possible. In contrast to their patients, 8 out of 10 of our patients presented with locally recurrent disease after a first resection of the RAASB, most of them even locally very advanced with a tumor extension classified as III (still limited to the ipsilateral chest wall) or IV (far extended beyond ipsilateral chest wall, see Figure 1 in [5]). In such situations standard treatment recommendations are clearly missed but it is obvious, that an additional local treatment approach is needed. The presented treatment schedule is a promising option to further reduce the radiation dose recommended so far. This could reduce side effects in patients without compromising local control. In addition, this treatment could be an option for elderly patients with reduced physical condition, as they may not be able to tolerate extended radical surgery or aggressive systemic therapy. However, due to total number of cases in both publications [1,5] further research is needed.

Angiosarcomas of the breast, whether it be PAS or RAAS, remain a very challenging disease to treat. Communications of different treatment approaches, e.g., extended surgical procedures, NAC or AC, adjuvant RT, or re-RT with/without hyperthermia reflect the whole spectrum of attempts to improve local and general tumor control. A combination of all four treatment modalities may be the key solution to this disease, with adaptions to each respective case. Moderate, regional hyperthermia is not only a radiosensitizer but also a chemosensitizer. In a randomized phase III trial comparing regional hyperthermia combined with chemotherapy to chemotherapy alone, the addition of hyperthermia significantly improved not only local tumor control but also overall survival in patients with high-risk soft tissue sarcomas [6]. One of the largest multicenter studies retrospectively analyzed the outcome of angiosarcoma patients concluding, that multimodal treatment may be beneficial for angiosarcomas [7], but the role of NAC should be further explored.

To conclude: prospective, multi-institutional studies are clearly needed. How to proceed from here? A first step could be to establish an international-wide patterns of care study with participation of all interested centers. If this works, and a consensus is found, a prospective evaluation of different treatment concepts including systemic treatments and local therapies can be started. The potential of additional loco-regional hyperthermia as a radio-, chemo- and immune-sensitizer could then be integrated into these curative strategies. We are interested in remarks and suggestions from the scientific community about further strategies to improve outcomes and the level of treatment recommendations.
